# The activities of the young professionals EPMA in the Czech Republic

**DOI:** 10.1186/1878-5085-5-S1-A21

**Published:** 2014-02-11

**Authors:** Jiri Polivka Jr, Pavla Sadilkova, Michaela Miklikova, Ondrej Topolcan, Milena Kralickova, Jiri Polivka

**Affiliations:** 1 Department of Histology and Embryology, Faculty of Medicine in Pilsen, Charles University in Prague, Czech Republic; 2 Biomedical Centre, Faculty of Medicine in Pilsen, Charles University in Prague, Czech Republic; 3 Department of Nuclear Medicine, Immunoanalytic Laboratory, University Hospital Pilsen, Czech Republic; 4 Department of Neurology, Faculty of Medicine in Pilsen, Charles University in Prague and University Hospital Pilsen, Czech Republic

## 

Personalized medicine is a perspective novel model of medical care being tailored to individual patients in whatever ways possible. Personalized medicine combines information of new “omics” with new preventive and therapeutic strategies, which are more efficient, safe and cost-effective. Personalized medicine affects the health care system and its decisive role in the near future is evident. The needs are the application of these principles into the clinical practice. The cardinal target is the education of physicians. The education is thought to be most effective in young physicians and medical students. The section of young specialists representing the EPMA in the Czech Republic was created at the Faculty of Medicine in Pilsen (Plzen) of the Charles University in Prague. The activities are coordinated in the close cooperation with the EPMA delegate in the Czech Republic professor Topolcan (University Hospital Pilsen), vice-dean of the Faculty of Medicine in Pilsen and the head of the Department of Neurology, University Hospital and Faculty of Medicine in Pilsen. One of the first activities was the “Personalized Medicine Questionnaire”, which was addressed to medical students in Pilsen. The results showed lacking knowldge in the principles of personalized medicine and the will of education in this field. On that account several educational activities were realized in our institution (workshops, conferences, seminars, new optional courses) addressed to medical students and young physicians. The presentation of special topics on the Czech website of Personalized medicine is our next step in the support of education and is widely accessible. The courses during the school year are very busy and there is not time enough for the more complex personalized medicine interdisciplinary sessions and discussions. For this reason we are working together on the organization of the first course of the “Summer School of Personalized Medicine in Pilsen 2014”, the first event in the Czech Republic on this topic. During two weeks the theoretical background and clinical application into the variety of the fields of medicine will be presented and discussed. The Summer School will be open to the students of medicine of Charles University and of other medical schools in the Czech Republic and hopefully for medical students abroad.

Supported by the project ED2.1.00/03.0076 from European Regional Development Fund and by Ministry of Health, Czech Republic - conceptual development of research organization (Faculty Hospital Pilsen - FNPl, 00669806)

